# Purification and characterization of nitrile hydratase of mutant 4D of *Rhodococcus rhodochrous* PA-34

**DOI:** 10.1007/s13205-012-0081-5

**Published:** 2012-08-24

**Authors:** Amit Pratush, Amit Seth, Tek Chand Bhalla

**Affiliations:** Department of Biotechnology, Himachal Pradesh University, Summer Hill, Shimla, 171005 India

**Keywords:** Characterization, Mutant, Purification, *Rhodococcus rhodochrous* PA-34

## Abstract

Nitrile hydratase (NHase; E.C. 4.2.1.84) has been purified and characterized using ammonium sulfate precipitation, ion exchange chromatography and gel filtration chromatography from the mutant 4D of *Rhodococcus rhodochrous* PA-34. The SDS-PAGE and MALDI-TOF analysis of the purified enzyme revealed that it is dimmer consisting of α- and β-subunits with a molecular mass of 25 and 30 kDa, respectively. The *K*_m_ and *V*_max_ values were 102 mM and 350.8 μmol/min/mg using 3-cyanopyridine as substrate. The purified NHase was stable in higher concentration of potassium ions and in acidic pH 5.5 as compared to NHase of the wild *R. rhodochrous* PA-34. The analysis of the N-terminal amino acid sequence of this enzyme revealed that this enzyme has 90 % homology with the high molecular weight nitrile hydratase of *R. rhodochrous* J1.

## Introduction

The nitrile metabolism in microorganisms mainly involves nitrilase, nitrile hydratase and amidase enzymes that convert nitriles to amides or acids (Asano et al. 1980; Bhalla et al. 1992; Yamada and Kobayashi 1996). This enzyme is one of the first enzymes used in industry for the production of a commodity chemical, i.e., Acrylamide (Raj et al. 2006, 2007; Prasad et al. 2007, 2009). Besides this a number of commercially important products such as nicotinamide, pyrazinamide, thiophenamide etc. have also been synthesized from the nitriles using this enzyme (Raj et al. 2006, 2007; Prasad et al. 2007, 2009; Kobayashi et al. 1992). Yet, there are very few organisms whose nitrile hydratase has shown promise for industrial application (Raj et al. 2006, 2007; Prasad et al. 2007; Kobayashi et al. 1992). The genetically modified organisms or mutants generated through chemical or physical mutagenesis having higher activity have not been either generated or perfected for the large scale applications in the conversion of nitriles to corresponding amides (Pratush et al. 2010).

*Rhodococcus rhodochrous* PA-34 has been reported as a potential organism that can convert acrylonitrile, butyronitrile and 3-cyanopyridine to corresponding amides at a high concentration (Raj et al. 2006, 2007; Prasad et al. 2007; Bhalla and Kumar 2005). A mutant 4D has been generated by chemical mutagenesis of *R. rhodochrous* PA-34 using MNNG that exhibited twofold increase in its nitrile hydratase activity for the conversion of 3-cyanopyridine to nicotinamide (Pratush et al. 2010). In this communication, we report the purification and characterization of nitrile hydratase of mutant 4D of *R. rhodochrous* PA-34.

## Materials and methods

### Chemicals

Nicotinamide and 3-cyanopyridine used in the present study were purchased from Sigma and Alfa Aesar, respectively. Other chemicals used in the present study were of analytical grade from various commercial sources.

### Microorganism

The mutant 4D of *R. rhodochrous* PA-34 generated earlier by Pratush et al. (2010) at the Department of Biotechnology, Himachal Pradesh University, Shimla, India.

### Culture, nitrile hydratase assay and estimation of nicotinamide

Mutant 4D cells were grown for the production of nitrile hydratase by following the procedure as detailed by Pratush et al. (2010). The NHase activity was assayed in 1 ml reaction containing 880 μl, 0.3 M Potassium phosphate buffer (pH 5.5), 20 μl (0.08 mg/ml) of enzyme and 100 μl of substrate 0.5 M 3-cyanopyridine following the method reported previously (Prasad et al. 2004).

### Purification of nitrile hydratase of mutant strain

All steps of purification were performed at 4 °C and 0.3 M potassium phosphate buffer pH 5.5 at 4 °C was used.

1. Homogenization of resting cells of *R. rhodochrous* PA-34 mutant 4D

The resting cells of mutant 4D were disrupted using bead beater (BSP make) having Zirconium beads (0.1 mm diameter) for 30 min in 10 disruption cycles at 4 °C. The resulting cell-free extract (CFE) was used as crude enzyme for subsequent NHase purification.

2. Ammonium sulfate fractionation of CFE

The cell-free extract was subjected to various % saturation concentration of ammonium sulfate (0–80 %). The fraction exhibiting maximum activity of NHase was termed as ASF and was taken for further purification of NHase.

3. Gel filtration of ASF

Ammonium sulfate fractionation was filtered through 0.45-μm filter and directly loaded on to pre-packed Sephacryl S-300 gel filtration column (16 mm diameter × 600 mm length) equilibrated with buffer. The gel filtration chromatography was performed using AKTA prime™ V2.00 at a flow rate 0.3 ml/min of elution buffer (0.3 M potassium phosphate buffer pH 5.5). NHase active fractions were subjected to SDS polyacrylamide gel electrophoresis (PAGE). The fractions exhibiting NHase activity were pooled. These pooled fractions were termed as GFF and used for further purification of NHase.

4. DEAE-ion exchange chromatography of GFF

The pooled filtered fractions of gel filtration (GFF) applied on an DEAE to an ion-exchange chromatography column (16 mm diameter × 100 mm length) equilibrated with 0.3 M potassium phosphate buffer pH 5.5. The column was eluted with a linear gradient of NaCl from 0.1 to 0.7 M in 0.3 M potassium phosphate buffer. NHase activity and protein concentration were estimated in each fraction. The fractions exhibiting single band on native-PAGE were pooled and termed as DEAEF (i.e., purified NHase).

### Characterization of purified NHase

#### Buffer, pH, temperature, substrate specificity and effect of metal ions and inhibitors on activity of purified NHase of mutant 4D

Various buffer systems (such as sodium phosphate and Tris–HCl and potassium phosphate buffer each 0.1 M pH 7.2) were used to select a suitable buffer to assay the activity of the purified NHase. The ionic strength of buffer (0.1–0.5 M potassium phosphate buffer) and pH (5.0, 5.5, 6.0, 6.5, 7.0, 7.5, 8.0, and 8.5) of selected buffer (potassium phosphate buffer) were optimized for NHase assay.

To determine temperature optimum of NHase of mutant 4D, the activity of this enzyme was determined at 10–80 °C. Thermal stability and substrate specificity profile of the purified mutant 4D NHase were tested after an interval of 20 min by subjecting purified NHase to 45, 55 and 65 °C for 8 h with different substrates, i.e., 2-cyanopyridine, 3-cyanopyridine, 4-cyanopyridine, butyronitrile, benzonitrile and acrylonitrile, respectively.

The effects of metal ions (AgNO_3_, CaCl_2_, CdCl_2_, CoCl_2_, CuCl_2_, FeCl_2_, HgCl_2_, MgCl_2_ and MnCl_2_) and inhibitors (ammonium persulfate (APS), DTT, EDTA, hydroxylamine, iodoacetic acid, l-ascorbic acid, phenyl hydrazine, phenylmethanesulphonylfluoride, semicarbazide, sodium azide and urea) on NHase activity of mutant were investigated by pre-incubating the enzyme at 1 mM concentration of metal ions/inhibitors for 30 min at 55 °C and then the NHase activity was assayed.

### Determination of *K*_m_ and *V*_max_ of NHase

The *K*_m_ and *V*_max_ of purified NHase of mutant 4D was calculated by determining initial velocity (*v*) of NHase at various concentrations of 3-cyanopyridine.

### Determination of molecular mass of NHase N-terminal amino acid sequencing and MALDI-TOF analysis of purified NHase of *R. rhodochrous* PA-34 mutant 4D and its analysis

SDS and native-PAGE were carried out to determine the purity, molecular mass of NHase and its subunits by the method of Laemmli (1970). The N-terminal amino acid sequence of purified NHase of mutant 4D was done at the Institute of Microbial Technology (IMTECH), Sector 39A, Chandigarh (India). Matrix assisted laser dissociated ionization-time of flight (MALDI-TOF) analysis of purified NHase was done at Jawaharlal Nehru University, New Delhi (India). Multiple protein sequence alignment was carried out using Clustal W program (Thompson et al. 1997; Chenna et al. 2003).

## Results and discussion

### Purification of mutant 4D NHase

The disruption of 2.0 g of resting cells of mutant 4D (containing 11,240 U of NHase activity) released 119 mg of protein and 1,000 U NHase activity in CFE. The purification of NHase of mutant 4D from the CFE involved ammonium sulfate fractionation (ASF), gel filtration chromatography (GFF) on Sephacryl S-300, DEAE-ion exchange chromatography (DEAEF). NHase protein was precipitated at 30–40 % saturation of ammonium sulfate (ASF), and it contained 90.7 mg protein with specific activity of 9.2 U/mg proteins (Table 1). Ammonium sulfate fractionation (30–40 % saturation cut) resulted in onefold purification of enzyme (AFS) with an yield of 83 % of NHase activity (Table 1). The NHases of *R. rhodochrous* PA-34 (Prasad et al. 2009), *R. rhodochrous* J1 (low and high molecular weight both) (Wieser et al. 1998; Nagasawa et al. 1991) and *Rhodococcus* sp. strain YH3-3 (Kato et al. 1999), *Brevibacterium* R312 (Nagasawa et al. 1986) and *Pseudomonas chlororaphis* B23 (Nagasawa et al. 1987) were precipitated in the 30–70 % and 40–55 % cut off ammonium sulfate, respectively.

**Table 1:** Purification table of nitrile hydratase of mutant

Stages of purifications	Total protein (mg)	Specific activity (units)	Total activity (units)	Yield (%)	Purification (fold)
Crude sample (CFE)	119	8.4	1,000	100	–
Ammonium sulfate precipitation (ASF)	90.7	9.2	835	83	1.0
Gel filtration (S-300) (GFF)	45.4	15.2	690	82	1.7
DEAE-ion exchange (DEAEF)	9.6	45	432	62	3

**Fig. 1:**
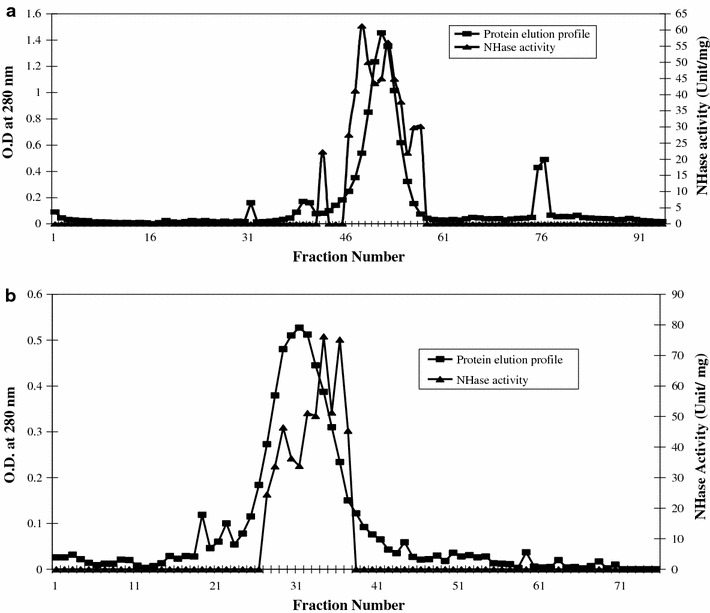
**a** Protein and NHase activity profile during gel permeation chromatography. **b** Protein and NHase activity profile by DEAE ion exchange chromatography

The dialysed protein of ASF was subjected to gel permeation chromatography using S-300 column (Fig. 1a). This step resulted in 1.7-fold of purification with an yield of 82 % having specific NHase activity of 15.2 U/mg protein. The fraction of GFF reached in NHase proteins was further loaded to the DEAE-ion exchange column (Fig. 1b), which resulted in the threefold purification of enzyme with a yield of 62 % and specific activity of 45 U/mg protein (Table 1). Earlier, the NHase of *R. rhodochrous* J1, *Agrobacterium tumefaciens* strain d3, *B. pallidus* Dac521, *P. chlororaphis* B23 and *Brevibacterium* R312 were purified by employing gel permeation chromatography techniques (Wieser et al. 1998; Nagasawa et al. 1986, 1987; Bauer et al. 1998; Cramp and Cowan 1999).

### Characterization of NHase of mutant 4D

**Fig. 2:**
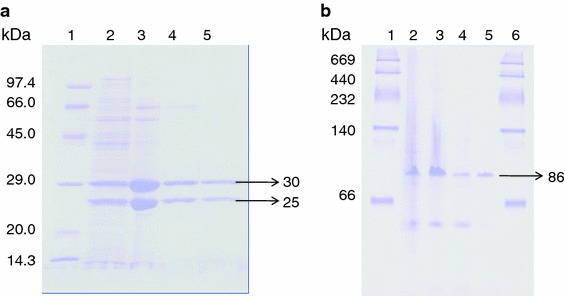
**a** Sodium dodecyl sulfate-polyacrylamide gel electrophoresis of NHase of mutant 4D at various steps of purification. SDS-protein molecular weight markers in kDa = kilo Dalton (*lane1*), cell-free protein extract (*lane2*), ammonium sulfate precipitation sample (*lane3*), gel filtration chromatography samples (*lane4*) and DEAE-ion exchange chromatography sample (*lane5*) SDS PAGE of purified NHase of mutant. **b** Native-PAGE analysis of purified NHase of mutant 4D, *lane1* was loaded with following molecular mass standards: thyroglobulin (660 kDa), ferritin (440 kDa), catalase (232 kDa), lactate dehydrogenase (140 kDa) and albumin (66  kDa). Cell-free extract (*lane2*), ammonium sulfate fraction (*lane3*) and gel permeation column chromatography fraction (*lane4*) DEAE-ion exchange column chromatography fraction (*lane5*)

**Fig. 3:**
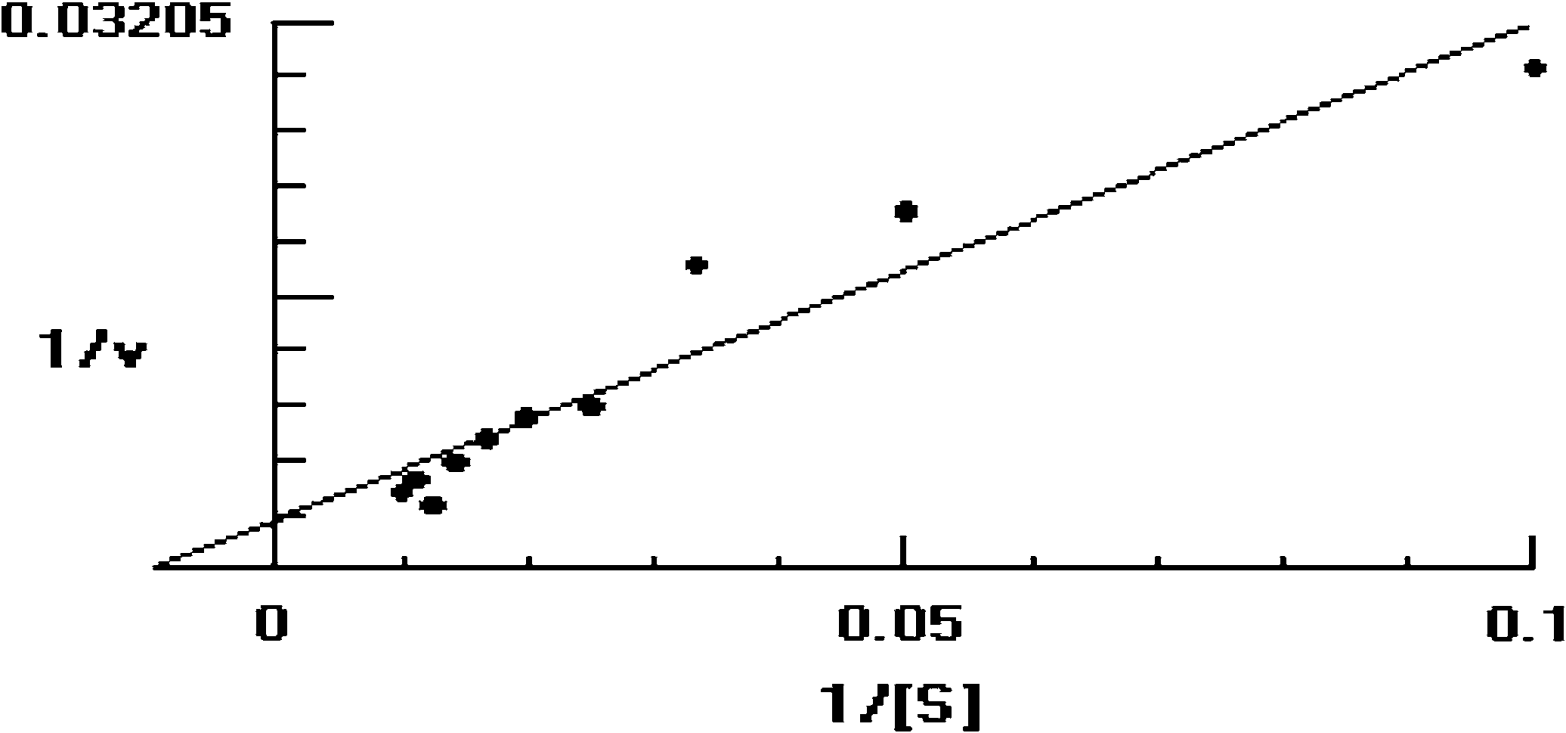
Lineweaver Burk plot of purified NHase of mutant

**Fig. 4:**
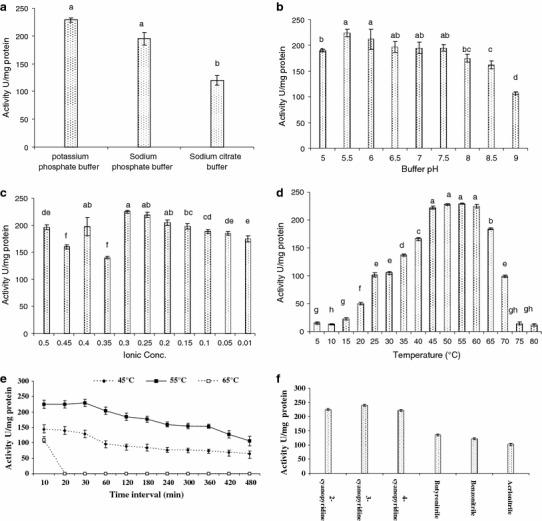
**a** Buffer system optimization for purified NHase of mutant. **b** Ionic strength optimization of buffer for purified NHase of mutant. **c** Buffer pH optimization for purified NHase of mutant. **d** Reaction temperature optimization for purified NHase of mutant. **e** Thermostability profile of purified NHase of mutant at 45, 55 and 65 °C. **f** Effect of different substrates on purified NHase of mutant

The purified NHase consisted of two polypeptides one comprising 25 kDa (α-subunit) and other was of 30.6 kDa (β-subunit) (Fig. 2a). It means that this enzyme is also constituted by two different polypeptides (α- and β-subunits) similar to the earlier reported NHases (Prasad et al. 2009; Wieser et al. 1998; Nagasawa et al. 1986). Native PAGE of purified NHase revealed a single band of 86 kDa (Fig. 2b). This indicated that the functional NHase might be constituted by one α- and two β-subunits (i.e., αβ_2_). The molecular mass of nitrile hydrates of different organisms varies from species to species, i.e., *R. rhodochrous* J1, *R. rhodochrous* sp. N774 and *Cornybacterium* sp. C5 have 59, 70 and 61 kDa, respectively (Kobayashi et al. 1991; Endo and Watanabe 1989; Yamamoto et al. 1992). The *K*_m_ and *V*_max_ values for the purified NHase of mutant 4D were 102 mM and 350.8 μmol/min/mg, respectively, using 3-cyanopyridine as substrate (Fig. 3), whereas *K*_m_ and *V*_max_ reported from other sources such as *R. rhodochrous* PA-34, L and H-NHase of *R. rhodochrous* J1 were 167 mM and 250 μmol/min/mg, 0.30 mM and 579 μmol/min/mg and 200 mM and 370 μmol/min/mg using 3-cyanopyridine as substrate, respectively (Prasad et al. 2009; Wieser et al. 1998). These results indicated that this enzyme had higher affinity for 3-cyanopyridine and had higher *V*_max_ in comparison to its wild strain. Among the three types of buffers tested, maximum NHase activity (225 U) was rerecorded in 0.3 M potassium phosphate buffer (Fig. 4a). Below and above 0.3 M concentration of the buffer the activity of NHase drastically decreased (Fig. 4b). The mutant NHase was found to be stable in acidic conditions, i.e., pH 5.5 (Fig. 4c). These results are drastically different from the earlier reports of Banerjee et al. (2002). Moreover, this NHase exhibited higher concentration of potassium ions, i.e., 0.3 M (Fig. 4b), whereas other reported NHases showed higher activity at 0.1 M potassium phosphate buffer (Raj et al. 2006; Prasad et al. 2009). The optimum temperature for assay of NHase activity turns out to be 55 °C (Fig. 4d), whereas it is 40 °C for *R. rhodochrous* PA-34 and *R. rhodochrous* J1 (Prasad et al. 2009; Wieser et al. 1998). Most of the nitrile hydratases have exhibited maximum activity near ambient temperature between 20 and 35 °C (Banerjee et al. 2002). The thermophilic NHases of *Bacillus* RAPc8 and *Pseudonocardia thermophila* showed maximum activity at 60 °C (Yamaki et al. 1997; Pereira et al. 1998).

The NHase of mutant 4D showed its maximum activity at 55 °C and it remained stable up to 5 h at this temperature, whereas the enzyme completely lost its activity at 65 °C (Fig. 4e). However, a thermophilic NHase from *B. pallidus* Dac 521 had a half-life of 0.85 h at 50 °C. The NHase from mutant 4D was much more stable than earlier reported NHase at higher temperature (Banerjee et al. 2002). NHase of mutant 4D showed highest activity with 3-cyanopyridine (Fig. 4f). The purified NHase of mutant 4D, showed almost complete loss of activity in the presence of metal ions. The NHase activity was inhibited up to 65.5–73.7 % by MgCl_2_ and hydroxyl amine, respectively. An inhibition of 30.5 % in NHase activity was observed in the presence of PMSF in reaction (Table 2). The loss of activity of NHase of mutant 4D in the presence of metal ions indicated the involvement of Cys residue at its active site, which might have got complexed with metal ions leading to inactivation of the enzyme.

**Table 2:** Effect of metal ions and compounds on purified nitrile hydratase of mutant

Metal ion/inhibitor (1 mM)	Relative activity (%)
None	100
AgNO_3_	0.266
CaCl_2_	77
CdCl_2_	61.8
CoCl_2_	74.8
CuCl_2_	0.824
FeCl_2_	0.74
HgCl_2_	0.04
MgCl_2_	34.5
MnCl_2_	73.5
Ammonium persulfate	42.5
Dithiothreitol	57.13
EDTA	71.3
Hydroxylamine	26.3
Iodoacetic acid	71.23
l-Ascorbic acid	72.6
Phenyl hydrazine	8.0
PMSF	69.5
Sodium azide	77.3
Urea	83.8

The N-terminal sequence of β- and α-subunits of mutant 4D NHase showed a significant (94 %) change in α-subunit when compared with the N-terminal sequence of α-subunit of wild *R. rhodochrous* PA-34, whereas the N-terminal sequence of β-subunit of mutant showed 90 % homology with the β-subunit of wild (Table 3). MALDI-TOF analysis revealed that purified NHase of mutant 4D had 90 % homology with the high molecular weight NHase of *R. rhodochrous* J1 (Kobayashi et al. 1991). The N-terminal and MALDI-TOF analysis of mutant 4D NHase protein revealed a significant change in the amino acid sequence of α- and β-subunits of wild strain and it exhibited high homology with H-NHase reported earlier from *R. rhodochrous* J1 (Kobayashi et al. 1991). These studies suggest that the wild strain *R. rhodochrous* PA-34 produces the L-NHase (low molecular weight nitrile hydratase), whereas the *R. rhodochrous* PA-34 mutant 4D produces H-NHase generated through chemical mutagenesis using MNNG. The chemical mutagenesis has switched off the expression of L-NHase gene and switch on the expression of H-NHase gene. However, further studies on cloning and sequencing of NHase operon of the wild and mutant 4D strains of *R. rhodochrous* PA-34 are needed further to explore this aspect at molecular level.

**Table 3:** N-terminal sequence of β- and α-subunits of mutant 4D and wild *Rhodococcus rhodochrous* PA-34

Subunit name	N-terminal sequence
β-subunit of mutant	M D G F H D T G N M
β-subunit of wild	M D G I H D L G G R
α-subunit of mutant	T E H V N K Y T E A
α-subunit of wild	T A H N P V Q G K L

## Conclusion

The NHase of *R. rhodochrous* PA-34 of mutant 4D produces high molecular nitrile hydratase with better thermal, ionic and acidic pH stabilities as compared to earlier reported nitrile hydratases including the NHase of wild strain.
